# Regulatory Effect of Osteocytes on Extramedullary and Bone Marrow Adipose Tissue Development and Function

**DOI:** 10.1007/s11914-024-00871-5

**Published:** 2024-04-16

**Authors:** Beata Lecka-Czernik, Mohd Parvez Khan, Joshua Letson, Sudipta Baroi, Amit Chougule

**Affiliations:** 1 Department of Orthopaedic Surgery, Center for Diabetes and Endocrine Research, University of Toledo, Toledo, OH 43614 USA; 2 Harvard University, School of Dental Medicine, Boston, MA 02115 USA; 3 University of Michigan, Ann Arbor, MI 48109 USA

**Keywords:** PPAR gamma, PPAR alpha, Sclerostin, Adipocyte, Osteocyte

## Abstract

**Purpose of Review:**

This review summarizes evidence on osteocyte support of extramedullary and bone marrow adipocyte development and discusses the role of endogenous osteocyte activities of nuclear receptors peroxisome proliferator-activated receptor gamma (PPARG) and alpha (PPARA) in this support.

**Recent Findings:**

PPARG and PPARA proteins, key regulators of glucose and fatty acid metabolism, are highly expressed in osteocytes. They play significant roles in the regulation of osteocyte secretome and osteocyte bioenergetics; both activities contributing to the levels of systemic energy metabolism in part through an effect on metabolic function of extramedullary and bone marrow adipocytes. The PPARs-controlled osteocyte endocrine/paracrine activities, including sclerostin expression, directly regulate adipocyte function, while the PPARs-controlled osteocyte fuel utilization and oxidative phosphorylation contribute to the skeletal demands for glucose and fatty acids, whose availability is under the control of adipocytes.

**Summary:**

Bone is an inherent element of systemic energy metabolism with PPAR nuclear receptors regulating osteocyte-adipocyte metabolic axes.

## Introduction

Osteocytes constitute 90–95% of bone cells and are localized in the mineralized bone compartment. Some estimates suggest that their number in the skeleton is comparable to half of the brain neuron number, reaching approximately 40 billion cells in the adult human skeleton [1]. Therefore, even subtle changes in osteocyte metabolism or production of secreted proteins may have significant local and systemic effects. Osteocytes are considered the major endocrine cells in the bone. They orchestrate bone remodeling by producing factors regulating, in a coordinated manner, bone formation by osteoblasts and bone resorption by osteoclasts. Osteocytes produce sclerostin, which inhibits bone formation by inhibiting WNT signaling in osteoblasts, and receptor activator of nuclear factor kappa beta ligand (RANKL), which increases bone resorption by osteoclasts. Besides sclerostin and RANKL, they also produce a number of cytokines and growth factors which are released to the bone cavity as well as to circulation modifying the bone marrow environment and regulating the function of distal organs, respectively.

Osteocyte role in the regulation of bone mass is related to their response to biomechanical signals. Interestingly, it has been recently demonstrated that rodent homeostatic regulation of body weight is controlled by mechanosensing activity of osteocytes [2] implicating that osteocyte skeletal and energy metabolism activities may share common mechanisms. This particular study concluded that osteocytes may act as gravitostat counteracting body weight gain in obese animals by reducing food intake independently of leptin, sclerostin, and lipocalin 2 [2, 3].

On the other hand, sclerostin protein, whose expression in osteocytes is under the control of peroxisome proliferator-activated receptor gamma (PPARG) [4••, 5••], has been signified for its direct effect on adipocyte differentiation and function. In addition, several recent studies have shown that osteocyte contribution to global energy metabolism is under the control of PPARG and PPARA nuclear receptors, two major regulators of metabolism and adipose tissue function [4••, 5••, 6••, 7•]. In this review, we will focus on the regulation of adipocyte development and function from the perspective of PPARG and PPARA activities in osteocytes and discuss possible consequences of this regulation for bone aging and cancer metastasis.

## Osteocyte Secretome Including Sclerostin Regulates Adipocyte Differentiation and Function

Osteocyte contribution to the regulation of body adiposity is supported by a number of studies. One of the earliest, by Sato et al., demonstrated that osteocytes play a central role in the maintenance of peripheral white adipose tissue (WAT) mass and in support for lymphopoiesis [8]. It was shown that osteocyte ablation in mice leads to loss of epidydimal WAT and severely compromised B and T lymphocyte development in the bone marrow and thymus. This phenotype was reverted only by replenishing of osteocytes in the bone but not by manipulation with either circulating humoral factors, or hypothalamic nuclei regulating peripheral fat mass, or supplying T cell progenitors. Unfortunately, marrow adiposity was not accessed in this model. Nevertheless, with a conclusion that osteocytes are essential for control of fat tissue maintenance in the whole organism and regulation of microenvironment supporting lymphopoiesis, this study significantly contributed to the development of a new area of investigation on osteocyte-adipocyte communication axes.

Osteocytes are a major source of sclerostin, a secreted glycoprotein which is a pharmacologic target to treat osteoporosis. Besides the bone, kidney and vascular smooth muscle are the other sources of sclerostin in circulation. Due to its inhibition of WNT signaling, sclerostin affects the lineage allocation of peripheral mesenchymal stem cells (MSC) and marrow skeletal stem cells (SSC) by inhibiting osteoblast and favoring adipocyte differentiation. In the 3T3-L1 cell line, a model of peripheral white adipocyte differentiation, sclerostin enhanced adipocyte-specific gene expression and lipid accumulation [9]. It antagonized WNT3a’s inhibitory effect on adipogenesis via a mechanism involving TAZ, a transcriptional modulator of canonical WNT signaling and mesenchymal cell lineage allocation [10]. Similarly, sclerostin inhibited WNT signaling in marrow mesenchymal progenitors or SSC which resulted in augmented adipocytic differentiation of these cells [11•].

Subsequently, these and other *in vitro* and *ex vivo* observations were confirmed in animal models. In mice, sclerostin deficiency (due to genetic manipulation or a decrease in circulating sclerostin levels using sclerostin-neutralizing antibodies) led to a decrease in the peripheral WAT mass, both epididymal and inguinal, and a reduction in bone marrow adipose tissue (BMAT) volume, but no changes in interscapular brown adipose tissue (iBAT) [5••, 11•, 12]. Mechanistically, it has been suggested that sclerostin increases differentiation of subpopulation of CD45-:Sca1 + :PDGFRα + adipoprogenitors residing in the vascular stroma of extramedullary WAT. In the absence of sclerostin, the differentiation of CD45-:Sca1 + :PDGFRα + progenitors to adipocytes was blocked with a simultaneous increase in canonical WNT pathway activity [13].

While the mechanism by which sclerostin increases adipocyte differentiation seems to be at least partially explained, the mechanism by which sclerostin regulates adipose tissue metabolic activity is unclear and debatable since some studies contradict others in their conclusions. In addition to decreased fat mass, Kim et al. showed that global ablation or pharmacologic inhibition of sclerostin led to improved insulin sensitivity and protection from diet-induced obesity that was associated with the “beiging” of peripheral WAT [13]. This correlated with increased fatty acid oxidation and their utilization by adipocytes as well as decreased *de novo* fatty acid synthesis, whereas sclerostin overproduction led to adipocyte hypertrophy associated with a phenotype of decreased energy metabolism [12]. Similarly, disruption of the WNT signaling pathway by inactivation of the WNT co-receptor LRP5 in osteoblasts and osteocytes under control of osteocalcin promoter, decreased bone mass and increased body fat mass and circulating lipids with a concomitant decrease in lean mass and energy expenditure that was attributed to reduced fatty acid β-oxidation and utilization by bone [14].

In contrast, an analysis of mice with high sclerostin levels in circulation due to constitutive or inducible deletion of the stimulatory subunit of G-proteins (Gsα) in osteocytes showed progressive loss of WAT and acquisition of beige adipocyte characteristics in gonadal and inguinal fat depots [15]. It has been postulated that circulating sclerostin directly induces beige adipogenesis; however, treatment of Gsα-deficient mice with sclerostin-neutralizing antibodies only partially reverted this phenotype, opening a possibility that other factors produced by osteocytes deficient in Gsα are contributing to the phenotype of increased energy metabolism. It should be noted that in the model used in Fulzele et al.’s study, Gsα expression was also disrupted in skeletal muscle, even though no effects on body composition have been observed in animals with Gsα deletion specifically in muscle, however, beiging of fat was not examined in this model [16].

Clinical data brings additional perspectives to an association between sclerostin levels and adipose tissue mass. A cross-sectional study in the Age Gene/Environment Susceptibility-Reykjavik cohort showed that in older men but not women (mean age 79 years) sclerostin levels positively correlated with higher fat volume in vertebra and total fat mass [17]. A more recent European study performed on the cohort of postmenopausal women with and without fragility fractures demonstrated a lack of correlation between circulating sclerostin and marrow fat content in vertebra and in hip [18]. However, this study showed a negative correlation between total fat mass and levels of circulating sclerostin in postmenopausal women with fragility fractures, and a negative correlation with visceral adiposity in both groups. In contrast, two studies performed on cohorts of Chinese and Japanese postmenopausal women consistently showed a positive correlation between sclerostin levels in circulation and peripheral fat mass [19, 20]. Moreover, there was a positive correlation between sclerostin and low-density lipoprotein cholesterol and homocysteine levels indicating a link to metabolic diseases [20]. Similarly, in younger women (< 50 years old), high sclerostin levels correlated with obesity, but in contrast to older postmenopausal women, sclerostin correlated negatively with spine and femoral neck BMD and the trabecular bone score [17, 18, 21]. These clinical studies underscore the complexity of sclerostin and adipose tissue relationship and emphasize sex, age, and ethnicity as important gene-by-environment factors mediating this relationship.

## PPARG and PPARA Control Metabolic and Endocrine Activities of Osteocytes

While testing the effects of PPARG/PPARA selective modulators on bone, osteocytes were identified as major targets for their activities in bone. In contrast to full PPARG agonists such as rosiglitazone and pioglitazone, the SR10171 compound (with activity of an inverse PPARG agonist and a weak PPARA agonist) increased bone mass by targeting osteocytes, and specifically by decreasing levels of sclerostin [22]. Interestingly, both PPARG and PPARA are expressed at relatively high levels in femoral osteocytes, which are comparable to their expression in the muscle, and are significantly higher than their expression in femoral endosteal osteoblasts [4••, 6••]. In addition to other existing models, mice with deletion of either PPARG or PPARA in cells of osteocytic lineage (γOT^KO^ and αOT^KO^ mice, respectively) provide a glimpse on the role of these two nuclear receptors in osteocyte contribution to the regulation of extramedullary and bone marrow adipose tissue and global regulation of energy metabolism.

The examples of three following models of PPARG deficiency and one model of PPARA deficiency in cells of osteoblast/osteocyte lineage offer mechanisms by which osteocytes regulate adipose tissue. In the first model, deletion of PPARG under Dmp1-Cre promoter-driver resulted in mice with increased bone mass and high energy metabolism reflected in increased energy expenditure, lean phenotype, decreased glucose levels, enhanced insulin secretion and sensitivity, and protection from a high-fat diet-induced dysmetabolism [7•]. These features were accompanied by increased thermogenic capacity, browning/beiging of extramedullary adipose tissue, and improved fatty acid oxidation. It was concluded that these effects were independent of sclerostin. Instead, the authors identified BMP7 cytokine as a mediator of the metabolic effects [7•]. They demonstrated that BMP7 levels were increased in the circulation of mice with osteocytic deletion of PPARG and that administration of BMP-neutralizing antibodies partially corrected hypoglycemia in mice and *in vitro* effects on steatosis in hepatocytes and fatty acid oxidation in 3T3-L1 adipocytes.

The second model consisted of mice with PPARG deletion under the Ocn-Cre promoter-driver, which equally affects osteoblast and osteocytes [5••]. Similarly, as in Brun et al.’s model, these mice were characterized with increased energy metabolism and increased insulin sensitivity associated with extramedullary fat beiging. However, in a series of loss-of-function and gain-of-function experiments, Kim et al. showed that decrease in levels of circulating sclerostin causes fat beiging and leads to increased energy metabolism. They also confirmed that sclerostin expression is under positive control of PPARG [5••].

**Fig. 1:**
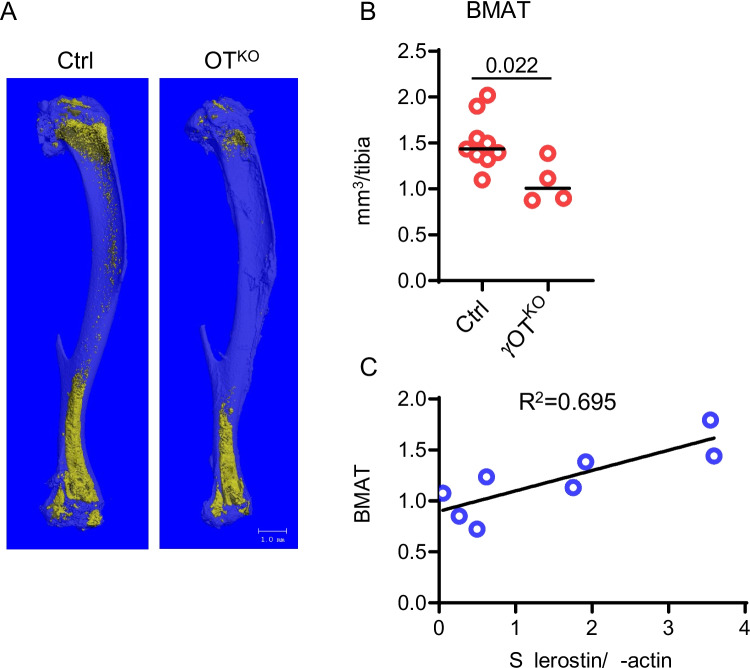
BMAT volume in tibia of Ctrl and γOT^KO^ mice. **A** MicroCT renderings of OsO_4_ stained tibia of 7-mo-old females. **B** Volumetric measurements of BMAT volume in 7-mo-old female tibia. **C** Pearson correlation of BMAT volume (determined by microCT) and sclerostin protein levels (determined by Western blot) in osteocytes of Ctrl and γOT^KO^ 6.5-mo-old male mice. From Baroi et al. (ref. 4)

The third model by Baroi et al., which was developed using identical tools as the Brun et al. model (crossing the same 10 kb Dmp1^Cre^ strain with Pparγ^fl/fl^ strain), showed only partial overlap with respect to the metabolic phenotype [4••, 23••]. Although γOT^KO^ mice were also characterized with high energy metabolism and a tendency to decreased fat mass, this phenotype was not associated with either extramedullary fat beiging, decreased glucose levels, or increased insulin sensitivity [23••]. Instead, the γOT^KO^ male mice were presented with apparent insulin resistance which correlated with a loss of insulin signaling in osteocytes. Moreover, PPARG has been identified as a positive transcriptional regulator of sclerostin expression via multiple PPRE sequences in the proximal and distal promoters of the *Sost* gene [4••, 23••]. Consistent with this, the γOT^KO^ mice have decreased BMAT volume in long bones which directly correlated with the level of sclerostin protein in the same bone [4••] (Fig. 1). However, sclerostin levels in circulation were not significantly affected. This may explain a lack of sclerostin effects on metabolism of extramedullary fat and indicates suboptimal penetration of phenotype in γOT^KO^ strain of mice. Thus, with the absence of osteocyte sclerostin-mediated endocrine effect on metabolism of extramedullary fat, this model unveils a new mechanism by which osteocyte metabolism contributes to the regulation of systemic energy metabolism [23••].

Detailed characterization of γOT^KO^ mice led to the conclusion that PPARG in osteocytes acts as a molecular brake of their metabolic activity. PPARG deficiency resulted in increased osteocyte bioenergetics manifested with increased mitochondrial activity, increased oxidative phosphorylation, increased ATP production, and increased fuel utilization, including glucose, fatty acids, and glutamine. Apparently, partial penetration of PPARG knock-out phenotype in this model allowed for the determination of osteocyte contribution to the systemic energy metabolism via their bioenergetics. The authors interpreted the high energy metabolism phenotype as a consequence of increased and dysregulated demands of osteocytes for fuel and increased mitochondrial activity [23••].

Similarly, mice deficient in PPARA (αOT^KO^) protein in cells of osteocytic lineage are characterized by relatively high energy metabolism, especially at younger ages, albeit due to different mechanisms [6••]. Deletion of PPARA did not increase osteocyte bioenergetics and ATP production in basal conditions; however, it increased mitochondrial respiration and spare respiratory capacity in response to stress. Most importantly, e/gWAT of αOT^KO^ mice has increased expression of fatty acid transporters and increased expression of *Prdm16* and *Ucp1* suggesting increased fatty acid metabolism and “beiging” of peripheral fat. When the energy metabolism quotient of αOT^KO^ mice was compared to mice with global PPARA deficiency (αKO), which also showed increased expression of “beige” markers in peripheral WAT, it was estimated that PPARA in osteocytes contributes up to 40% to the total energy production under PPARA control. It was concluded that increased energy metabolism in αOT^KO^ mice results rather from endocrine activities of osteocytes regulating peripheral fat metabolism, while increased energy metabolism observed in all three mouse models with PPARG deletion resulted from summation of increased osteocyte bioenergetics and osteocyte secretome activities leading to beiging of extramedullary fat.

In contrast to γOT^KO^, volume of BMAT in bone of αOT^KO^ mice was increased. This increase was not due to a change in the lineage commitment of SSC but rather due to the paracrine activities of PPARA-deficient osteocytes, whose conditioned media are unable to induce adipocytic gene expression in SSC as compared to PPARA-intact control osteocytes [6••]. Most importantly, sclerostin was not involved in this effect since PPARA did not affect its expression and its levels in circulation; however, WNT signaling cannot be excluded. It has been demonstrated that a lack of PPARA in osteocytes downregulates WNT, TGF-β, and Hippo pro-osteoblastic signaling, providing a possibility that additional to sclerostin osteocyte-specific factors are involved in regulation of marrow adiposity [6••].

It is important to consider that sclerostin may represent one of several adipocyte-inductive activities produced by osteocytes. Depletion of sclerostin from the conditioned media collected from a bone organ culture consisting of femoral cortical bone cleaned from periosteal and endosteal osteoblasts, reduced expression of pro-adipocytic markers in SSC by only 50% [6••]. Moreover, Pearson correlation coefficient between BMAT volume and sclerostin protein levels in femoral bone of WT and γOT^KO^ mice is much less strong (*R*^2^ = 0.695) than correlation coefficient between the levels of sclerostin and PPARG protein (*R*^2^ = 0.982). This points to other undiscovered factors which might regulate bone marrow and extramedullary adiposity. Among the suspects, DKK1 and other inhibitors of WNT pathway, as well as BMP signaling pathway, should be considered. This might be especially important for osteocyte role in supporting BMAT contribution to bone aging and cancer metastasis, as discussed below.

## Perspectives on Osteocyte Support for BMAT Role in Bone Aging and Cancer Metastasis

Among terminally differentiated bone cells, osteocytes and adipocytes have the longest lifespan as compared to high turnover of osteoblasts, osteoclasts, and hematopoietic cells. These two cell types have immense control of bone metabolism by directly orchestrating bone remodeling and/or modulating bone microenvironment. As long-lived cells, osteocytes and adipocytes undergo cellular senescence; the phase of their life hallmarked by production of signaling named senescence-associated secretory phenotype (SASP) that affects neighboring non-senescent cells and leads to the organ disfunction, which in bone is measured by unbalanced bone remodeling, loss of bone material properties, and fractures [24]. Osteocyte senescence either naturally, pathologically (e.g., diabetes), or pharmacologically (e.g., glucocorticoids) induced is gaining a lot of attention and a number of SASP proteins have been identified with a premise to prevent bone loss and fractures [25]. There are therapies in development targeting either SASP or senescent osteocytes and ongoing limited clinical trials are providing promising results [26, 27].

The role of bone marrow adiposity in cellular senescence and bone aging has been a subject of scientific investigation for the last few decades. As individuals’ age, there is a shift in the composition of bone marrow towards adipocyte rich manifested by increased BMAT volume [28]. Besides aging, other pathophysiological proceedings like obesity, type 2 diabetes, and osteoporosis as well as treatments with different pharmacological agents like thiazolidinediones (TZDs) and glucocorticoids have also been established to augment the bone marrow adiposity [29, 30]. Adipokines, including adipsin and inflammatory cytokines released from senescent marrow adipocytes, have been shown to contribute to senescence of bone cells and bone loss with aging [31]*.* Most recently, it has been discovered that bone marrow senescent cells synthesize many biologically active lipid molecules known as oxylipins that arise from the oxygenation of polyunsaturated fatty acids (PUFAs). Oxylipins together with glucocorticoids act as activators of PPARG activity and accelerate senescence of BMAT [32]. The senescent BMAT in turn exudes various cytokines or factors constituting SASP and including oxylipins, which in turn lead to secondary senescence in bone cells and bone marrow milieu [33]. Considering longevity of osteocytes and marrow adipocytes, it would not be surprising that these two cell types communicate and shape bone aging.

BMAT has been also linked to development of chronic inflammation and insulin resistance in type 2 diabetes (T2DM), in part via enhanced secretion of monocyte chemotactic protein-1 (MCP-1) [34]. Furthermore, BMAT is involved in the production and secretion of different hormones and adipokines which can influence insulin sensitivity and glucose metabolism, thereby dictating the development and progression of T2DM [35]*.* With evidence that T2DM induces osteocyte senescence and SASP production [24], there is a question on the role of marrow adipocytes in this equation.

BMAT plays a critical role in cancer metastasis to bone including breast, lung, and prostate cancers. Paget’s “seed and soil” hypothesis has been used to describe metastasizing cancer cell preference for sites within the body that can provide nutrients for their survival [36]. Bone offers an environment that is rich in nutritional soil for these cancer cells to feed from including marrow adipocytes. Besides nutrients, BMAT releases cytokines and adipokines that can interact with cancer cells and aid in their metastatic growth and survival [37–39]. In prostate cancer (PCa), BMAT releases chemokines CXCL1 and CXCL2, which induce osteoclastogenesis through CXCR2 signaling [40]. BMAT can play a critical role in PCa cell metabolism by inducing glycolytic enzymes via activation of HIF-1α and promoting a Warburg phenotype, the process by which cancer cells consume glucose for energy production [41]. Additionally, PCa cells were shown to induce lipolysis of marrow adipocytes providing nutrients for their growth and further demonstrating the paracrine interactions between BMAT and PCa cells. Similarly, in breast cancer metastasis, BMAT secrete many factors including IL-1β and leptin which were shown to enhance migratory phenotype of breast cancer cells [42, 43]. The question remains on the role of osteocytes and their secretome in modulating BMAT interaction with metastasizing cancer cells and whether osteocytes might be targeted to decrease cancer growth and bone destruction.

## Conclusions

Human and animal studies leave no doubt that the skeleton plays an important role in the regulation of systemic energy metabolism in physiological and pathological conditions. Although obvious but often not fully appreciated, bone demands fuel as it is one of the largest organs in our body. There is mounting evidence that bone endocrine activities regulate systemic energy metabolism by modulating functions of extramedullary and marrow adipocytes. Notably, osteocytes’ role in regulation of energy balance is gaining increased attention due to not only their quantity but also their bioenergetics and endocrine activities. Remarkably, osteocyte function is under control of two nuclear receptors, PPARG and PPARA, which are essential for regulation of glucose and fatty acid metabolism, and adipose tissue function. These provide new means for consideration of pharmacological intervention to treat skeletal and metabolic pathologies, simultaneously.

## Data Availability

No datasets were generated or analysed during the current study.
